# Occupational-Related Contact Dermatitis: Prevalence and Risk Factors Among Healthcare Workers in the Al'Qassim Region, Saudi Arabia During the COVID-19 Pandemic

**DOI:** 10.7759/cureus.10975

**Published:** 2020-10-15

**Authors:** Omar B Alluhayyan, Bashair K Alshahri, Abdulrahman M Farhat, Sulaiman Alsugair, Jihan J Siddiqui, Khaled Alghabawy, Ghaida B AlQefari, Waleed O Alolayan, Izzat A Abu Hashem

**Affiliations:** 1 Medicine, Qassim University, Qassim, SAU; 2 Medicine, Imam Abdulrahman bin Faisal University, Khobar, SAU; 3 Medicine, Sulaiman Al Rajhi University, Qassim, SAU; 4 Medicine, King Saud bin Abdulaziz University for Health Sciences, Riyadh, SAU; 5 Medicine, King Abdulaziz University, Jeddah, SAU; 6 Nursing, Alrras General Hospital, Qassim, SAU; 7 Family Medicine, Primary Health Care Center - As Sulimaniyah, Qassim, SAU

**Keywords:** covid-19, contact dermatitis, occupational disease, healthcare workers

## Abstract

Objective

This study aimed to estimate and investigate the prevalence and the risk factors implicated in contact dermatitis among healthcare workers in the Al'Qassim region, Saudi Arabia, during the COVID-19 pandemic.

Methodology

We conducted a cross-sectional survey among healthcare workers at hospitals in the Al'Qassim region. Data was collected using a standardized and validated Nordic Occupational Skin Questionnaire version 2002. We included 408 participants in the analysis.

Results

The majority of the respondents (66.7%) were females. The mean age of participants was 34 (SD: ±9) years. Most of the participants who reported contact dermatitis were nurses (58.6%). Direct patient care roles represent 78% of participants. Respondents who work 40-50 hours per week represent 61.5% of the sample. The most commonly recorded symptoms were dryness (92.9%), itchiness (50%), and redness (46.4%) of the skin. The most affected site was hand 93.5%. Hand cleanser was the commonest substance implicated in the worsening of the skin changes (59.2%). Protective glove material that worsens contact dermatitis, such as natural rubber/latex, represents 76% of responses. A significant association (p=0.001)was seen in the occurrence of contact dermatitis in those with a history of allergic eye symptoms (33.3%) and those without (58%). Participants with a mean age of 26.47 years were more prone to develop contact dermatitis (CI: 1.19-7.06; p=0.067). Pharmacists and interns had 3.69 and 4.90 times higher odds of having contact dermatitis (CI: 0.95-7.33; CI: 22.1; p=0.027; p=0.038, respectively). Those involved in patient education and research activities at work were 6.48 (p=0.017) and 20.51 (p=0.024) times likely to develop contact dermatitis (CI: 1.38-30.31; CI: 1.49-282.15, respectively).

Conclusions

We explored the prevalence and risk factors for occupational contact dermatitis among healthcare workers in Saudi Arabia. The prevalence of reported skin changes during the pandemic was 46.4%. Our study also showed that the risk factors of developing contact dermatitis include female gender, history of eye allergies, and young age group.

## Introduction

Coronavirus disease 2019 ( COVID-19) was detected in December 2019 in Wuhan, China. It is described as pneumonia of unknown cause that spreads from person to person [1]. The highly-Infectious virus spreads mainly by coughing or sneezing through droplets from the infected person, community transmission, and direct contact with contaminated objects. Appropriate use of personal protective equipment (PPE), personal hygiene, and social distancing reduce transmission rate [2]. World Health Organization (WHO) labeled COVID-19 as a global pandemic in March 2020, and all countries around the world were advised to take preventive measures to reduce the transmission of the virus [3]. In Saudi Arabia, the first case of COVID-19 was detected in a traveler on March 2nd, 2020 [4].

Frequent handwashing and prolonged use of PPE were observed among the healthcare workers ever since this pandemic started. Consequently, many skin diseases have emerged, such as contact dermatitis, pressure urticaria, and pressure injury. Many patients have complained about the worsening of their pre-existing skin conditions, including acne and seborrheic dermatitis [1]. Being a healthcare worker poses a high risk of developing occupational-related skin diseases [5]. Studies have shown that prolonged wearing of face mask can cause contact dermatitis, pressure urticaria, itching, acneiform eruption, and indentation. Protective caps cause scalp occlusion, which may exacerbate seborrheic dermatitis, folliculitis, and itching. According to reports, the nasal bridge is the most affected site due to the continuous, prolonged use of protective goggles. Healthcare workers frequently use latex gloves; their use contributes to blister formation, occlusion, and maceration, which may lead to contact dermatitis in certain cases. Also, excessive handwashing by detergents and disinfectants disrupts the hydrolipid barrier, which causes skin dryness and irritation. Scientists have attributed most of the skin manifestations in healthcare workers to hyperhydration, skin irritation, epidermal barrier disruption, and contact reactions that may result from prolonged use of personal protective equipment. These factors can also contribute to the worsening of pre-existing skin diseases [2].

The Health and Safety Executive describes occupational skin disease as any skin disorder which is caused or worsened by work or workplace activity. Occupational skin disease is considered the second most common occupational disease. It accounts for 25% of all lost workdays [5]. Several international studies reported the prevalence of occupational skin disease in healthcare workers between 17 and 55% [6]. Occupational contact dermatitis is responsible for 70-90% of all occupational skin diseases and can affect the patient's quality of life. The most common symptoms include itching, swelling, blisters, cracking, or skin flaking. A cross-sectional study was conducted over 12 months in Ethiopia to determine the prevalence of self-reported occupational-related contact dermatitis, indicating a prevalence of 31.5% among 422 healthcare providers [5]. 

Despite the problem recognition, there is insufficient data on the prevalence and risk factors for occupational-related contact dermatitis in Saudi Arabia during the COVID-19 pandemic. Therefore, we aimed to conduct a cross-sectional study to estimate the prevalence and investigate the risk factors for occupational-related contact dermatitis among the healthcare providers in the Al'Qassim region, Saudi Arabia, during the COVID-19 pandemic.

## Materials and methods

Study design, setting, and participants

This observational cross-sectional study was conducted from May to August 2020 in the Al'Qassim region of Saudi Arabia. The questionnaire was distributed among healthcare workers in the following four government hospitals in the Al'Qassim province: King Saud Hospital, Unaizah; Buraidah Central Hospital, Buraidah; Ar Rass General Hospital, Ar Rass; and Badai General Hospital, Badai. Also, an electronic self-administered survey was sent specifically to healthcare workers in Al'Qassim through social media. A consecutive sampling method was considered in this study. The sample size was calculated for an assumed prevalence of 50% and an absolute precision of 5% using the statistical formula for the cross-sectional survey design. A 95% confidence level was considered to obtain adequate power for analysis. Four hundred and eight participants were included in the study after adding a 6% non-response rate. The present study was approved by the regional bioethical committee of the Qassim region. Informed consent was gained from each patient to enroll in our study.

Inclusion and exclusion criteria

This study included healthcare workers (e.g., doctors, nurses, lab workers, other allied medical practitioners) who worked during the last four months in Al'Qassim province. All hospital administrative staff and people who had no direct contact with the patients were excluded from the study.

Data collection tools and techniques

Data was collected using a standardized Nordic Occupational Skin Questionnaire version 2002 [7]. The first part of the questionnaire was about demographic features (age, sex) and work-related questions (occupation, job hours per week, and direct or indirect contact with patients). In the second part, questions were modified to target any self-reported skin changes (i.e., dryness, redness, or itchiness) during the COVID-19 pandemic; this included affected areas of the body, number of episodes during the last two years, factors that worsen the condition (protective gloves and their type, antiseptics/disinfectants, face mask, and protective goggles), factors that improve the condition and the average time spent for hand washing and disinfecting. The last part obtained a history of any pre-existing allergic conditions and symptoms.

To ensure the quality of data obtained, we conducted a pre-test on a sample of healthcare providers before the actual data collection to test the instrument's validity and consistency, resulting in modifying some questions and misinterpretations. We also translated the questionnaire to the local language (Arabic) by experts to avoid misconceptions. 

Data management and analysis

The data were analyzed using SPSS version 25 (IBM Inc, Armonk, USA). Frequencies and percentages were calculated for categorical variables and mean ± standard deviation (SD) for continuous variables. Association between categorical variables was analyzed using the chi-square test (χ2). A p-value of ≤ 0.05 was considered statistically significant. The co-variables were compared and contrasted across the gender of participants and the presence of self-reported occupational contact dermatitis. A univariate logistic regression analysis was performed separately for all variables significantly associated with occupational contact dermatitis to explore the associations with the dependent variable. Furthermore, a multinomial logistic regression was also performed, including all variables significantly associated with occupational contact dermatitis to identify the correlates of different variables with self-reported occupational contact dermatitis. A cut of ≤ 0.05 p-value was set to evaluate the significance and odds ratios (OR) with a 95% confidence interval (CI) to establish the strength of associations. All results were summarized in tables and figures.

## Results

The participants' characteristics and clinical information are presented in Table 1. More than half of the respondents were female (66.7%; n=272). The mean age was 34 years (SD ± 9). Of all the healthcare workers recruited in the study, the greatest number of respondents were the nurses (58.6%; n=239), doctors (16.2%; n=66), pharmacists (8.3%; n=34), other occupations (7.1%; n=29), technicians (4.9%; n=20), and medical interns (2.9%; n=12). Around 78% of the respondents' major activity at work involved direct patient care (n=319), while the rest of the sample worked in administrative (11%; n=45), patient education (5.9%; n=24), indirect patient care (3.7%; n=15) and research (1.2%; n=5) roles. Out of all participates, 30% (n=53) indicated that they worked for less than 40 hours per week, 61.5% (n=251) worked 40-50 hours per week, 18.6% (n=76) worked 51-60 hours per week, and 6.9% worked overtime (more than 60 hours per week).

**Table 1:** Comparison of participants characteristics by gender in a sample of healthcare providers

		Total population	Gender				p-value
			Male		Female		
			n	%	n	%	
Total population = 408			136	33.3%	272	66.7%	N/A
Age (mean: 34 years; SD: ±9 years; median: 32 years)	20 to 29	158	33	20.9%	125	79.1%	<0.0001
	30 to 39	161	62	38.5%	99	61.5%	
	40 to 49	63	28	44.4%	35	55.6%	
	> 49	26	13	50.0%	13	50.0%	
Occupation	Doctors	66	50	75.8%	16	24.2%	<0.0001
	Nurses	239	21	8.8%	218	91.2%	
	Physiotherapists	8	5	62.5%	3	37.5%	
	Pharmacists	34	22	64.7%	12	35.3%	
	Technicians	20	14	70.0%	6	30.0%	
	Interns	12	5	41.7%	7	58.3%	
	Other	29	19	65.5%	10	34.5%	
What is your major activity at work?	Patient education	24	15	62.5%	9	37.5%	0.001
	Research	5	3	60.0%	2	40.0%	
	Administration	45	22	48.9%	23	51.1%	
	Direct patient care	319	90	28.2%	229	71.8%	
	Indirect patient care (patient supportive office)	15	6	40.0%	9	60.0%	
How many hours per week do you work in your main job (on average)?	< 40	53	28	52.8%	25	47.2%	0.001
	40-50	251	73	29.1%	178	70.9%	
	51-60	76	21	27.6%	55	72.4%	
	> 60	28	14	50.0%	14	50.0%	
Have you ever noticed any skin changes or symptoms, (i.e., dryness, redness, or itchiness) during COVID-19 pandemic?	No	219	87	39.7%	132	60.3%	
	Yes	189	49	25.9%	140	74.1%	0.003
Your skin changes initially started	Within the COVID 19 pandemic	134	36	26.9%	98	73.1%	0.693
	Before the COVID 19 pandemic	54	13	24.1%	41	75.9%	
What do you consider as the most important things in the workplace that worsen your skin changes?							
Protective gloves	No	109	29	26.6%	80	73.4%	0.847
	Yes	75	19	25.3%	56	74.7%	
Hand cleanser, soap	No	75	29	38.7%	46	61.3%	0.001
	Yes	109	19	17.4%	90	82.6%	
Antiseptics/disinfectants	No	96	27	28.1%	69	71.9%	0.511
	Yes	88	21	23.9%	67	76.1%	
Face mask	No	126	36	28.6%	90	71.4%	0.258
	Yes	58	12	20.7%	46	79.3%	
Protective goggles	No	180	47	26.1%	133	73.9%	0.960
	Yes	4	1	25.0%	3	75.0%	
None of the above	No	178	47	26.4%	131	73.6%	0.593
	Yes	6	1	16.7%	5	83.3%	
What do you consider as the most important things outside the workplace that worsen your skin changes?							
Frequent hand washing	No	90	32	35.6%	58	64.4%	0.001
	Yes	89	12	13.5%	77	86.5%	
Protective gloves	No	138	33	23.9%	105	76.1%	0.703
	Yes	41	11	26.8%	30	73.2%	
None of the above	No	168	40	23.8%	128	76.2%	0.349
	Yes	11	4	36.4%	7	63.6%	
Questions							
Does your skin changes improve when you are away from your normal work (for example weekends or longer periods)?	No	11	4	36.4%	7	63.6%	0.523
	Yes, sometimes	82	17	20.7%	65	79.3%	
	Yes, usually	78	20	25.6%	58	74.4%	
	Don't know	8	3	37.5%	5	62.5%	
How many times do you wash your hands during a usual working day before the COVID 19 pandemic?	0-5 times per day	105	51	48.6%	54	51.4%	<0.0001
	6-10 times per day	141	59	41.8%	82	58.2%	
	11-20 times per day	89	13	14.6%	76	85.4%	
	more than 20 times per day	73	13	17.8%	60	82.2%	
How many times do you wash your hands during a usual working day during the COVID 19 pandemic?	0-5 times per day	25	13	52.0%	12	48.0%	<0.0001
	6-10 times per day	99	50	50.5%	49	49.5%	
	11-20 times per day	110	34	30.9%	76	69.1%	
	more than 20 times per day	174	39	22.4%	135	77.6%	
How much time do you spend on each hand washing before the COVID 19 pandemic?	Less than 40 seconds	180	76	42.2%	104	57.8%	0.001
	40-50 seconds	175	51	29.1%	124	70.9%	
	More than 50 seconds	53	9	17.0%	44	83.0%	
How much time do you spend on each hand washing during the COVID 19 pandemic?	Less than 40 seconds	62	30	48.4%	32	51.6%	0.006
	40-50 seconds	226	76	33.6%	150	66.4%	
	More than 50 seconds	120	30	25.0%	90	75.0%	
How many times do you use disinfecting alcohol for hands in a usual working day before the COVID 19 pandemic?	0-5 times per day	145	74	51.0%	71	49.0%	<0.0001
	6-10 times per day	118	40	33.9%	78	66.1%	
	11-20 times per day	90	14	15.6%	76	84.4%	
	more than 20 times per day	55	8	14.5%	47	85.5%	
How many times do you use disinfecting alcohol for hands in a usual working day during the COVID 19 pandemic?	0-5 times per day	25	17	68.0%	8	32.0%	<0.0001
	6-10 times per day	107	50	46.7%	57	53.3%	
	11-20 times per day	121	33	27.3%	88	72.7%	
	more than 20 times per day	155	36	23.2%	119	76.8%	

**Figure 1:**
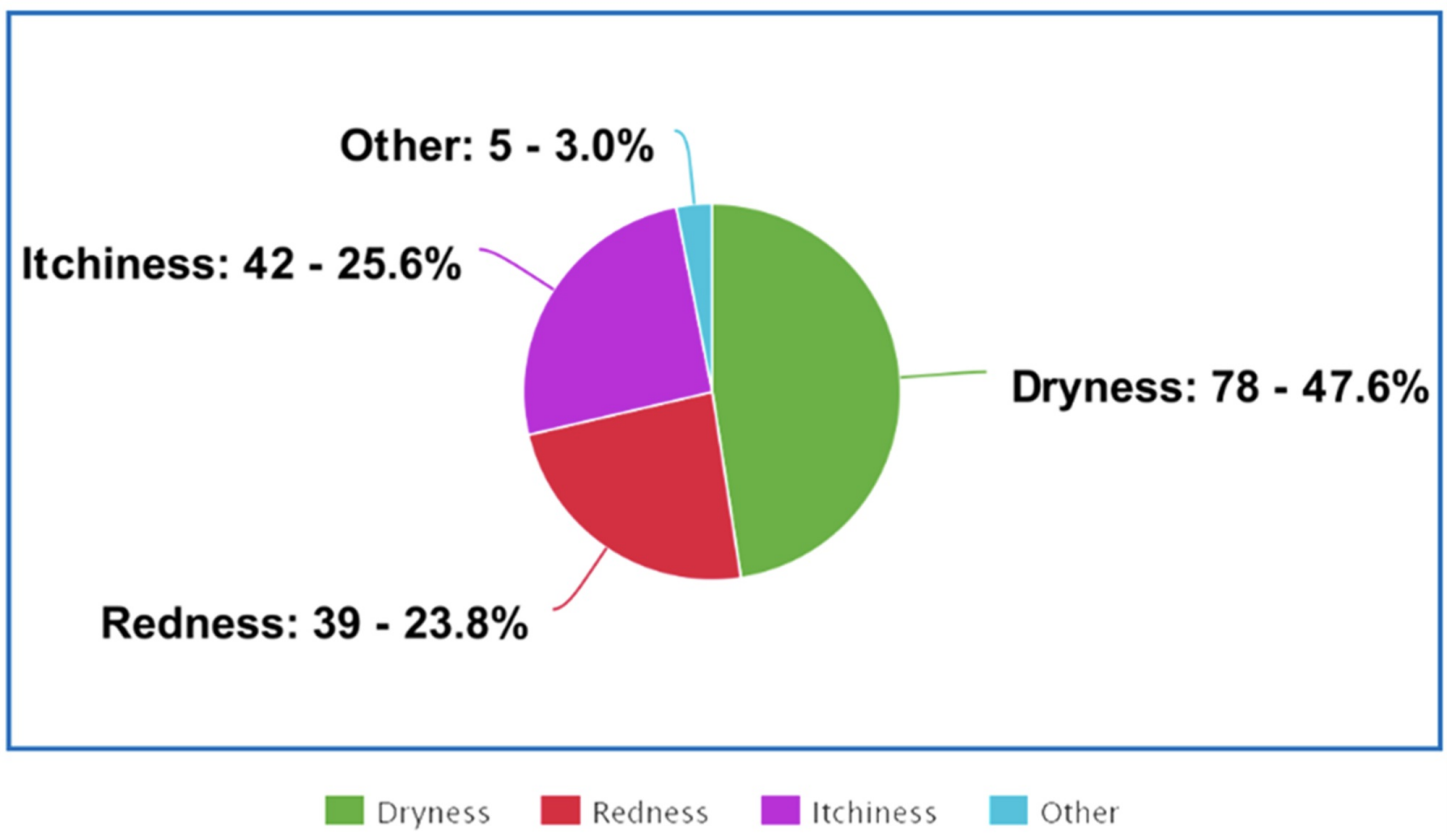
Distribution of the reported skin changes

**Figure 2:**
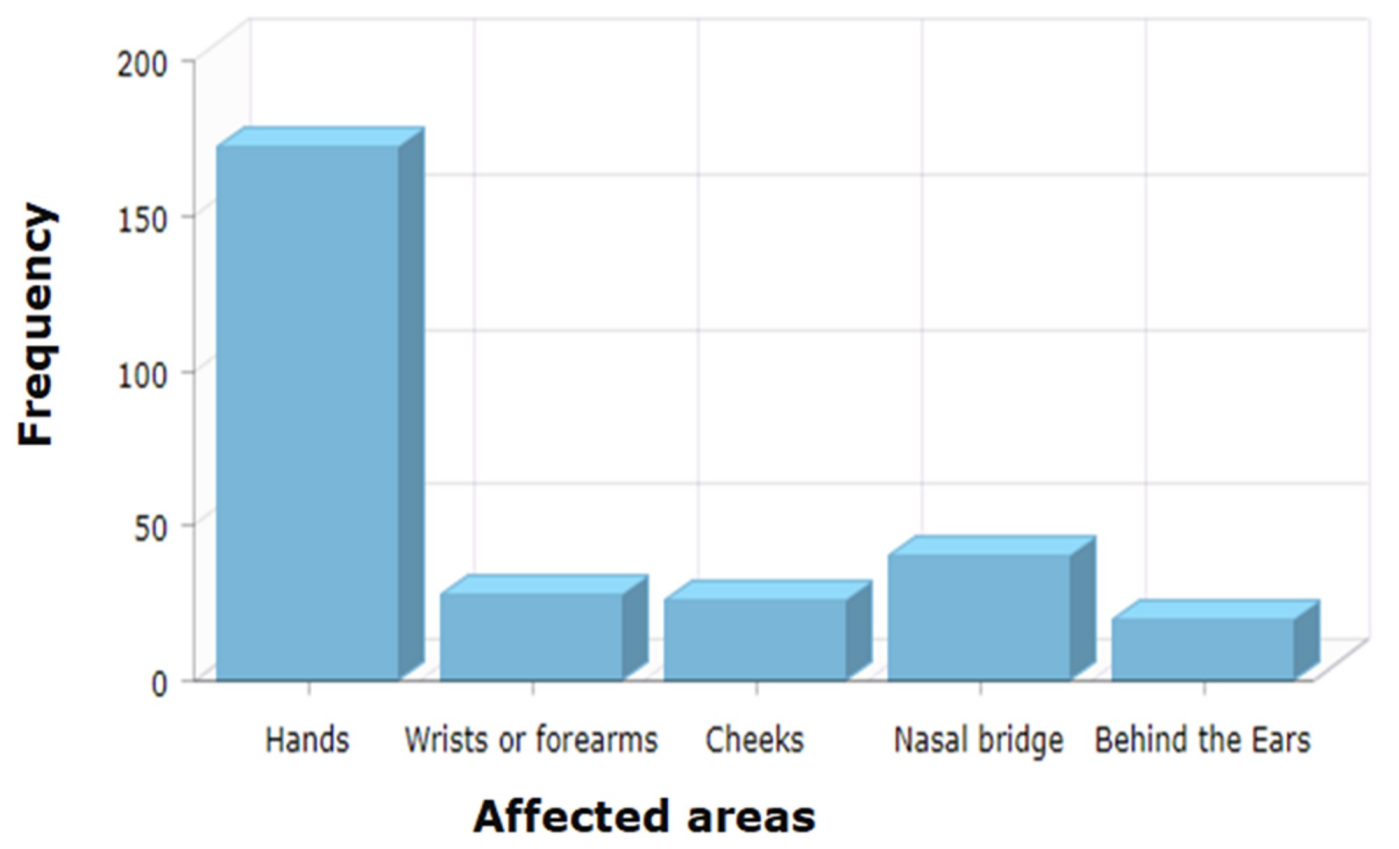
Frequency of the affected areas

Table 2 shows that the overall prevalence of self-reported skin changes (i.e., dryness, redness, or itchiness) during the COVID-19 pandemic was 46.3% (n=189). The most commonly reported types of skin changes were dryness (92.9%; n=78), itchiness (50%; n=42), and redness (46.4%; n=39), as indicated in Figure 1. Hands, nasal bridge, and wrists or forearms (excluding fronts of elbows) ranked as the three most commonly affected areas, as reported by 93.5% (n=172), 21.7% (n=40), and 15.2% (n=28) respondents respectively (Figure 2). The three most commonly reported factors in the workplace that worsen the participants' skin changes were hand cleanser/soap (59.2%; n=109), antiseptic/disinfectants 47.8%; n=88), and protective gloves (2.2%; n=75). Respondents reported that 76% (n=57) wear natural rubber/latex gloves, 16% (n=12) wear synthetic rubber gloves, and 12% (n=9) wear plastic gloves. A significant association (p=0.001) was seen in the occurrence of contact dermatitis between those who had a personal history suggestive of allergic eye symptoms (itching, watery, red, or swollen eyes, e.g., from pollen or animals) reported by 33.3% (n=23), and those who had not (58%; n=188).

**Table 2:** Prevalence of skin changes by multiple factors among a sample of healthcare providers

		Have you ever noticed any skin changes or symptoms, (i.e. dryness, redness, or itchiness) during COVID-19 pandemic?				p-value
		No		Yes		
		n	%	n	%	
Gender	Male	87	64.0%	49	36.0%	0.003
	Female	132	48.5%	140	51.5%	
Age	20 to 29	69	43.7%	89	56.3%	0.000
	30 to 39	86	53.4%	75	46.6%	
	40 to 49	46	73.0%	17	27.0%	
	> 49	18	69.2%	8	30.8%	
Occupation	Doctors	36	54.5%	30	45.5%	0.040
	Nurses	128	53.6%	111	46.4%	
	Physiotherapists	6	75.0%	2	25.0%	
	Pharmacists	13	38.2%	21	61.8%	
	Technicians	15	75.0%	5	25.0%	
	Interns	3	25.0%	9	75.0%	
	Other	18	62.1%	11	37.9%	
What is your major activity at work?	Patient education	9	37.5%	15	62.5%	0.021
	Research	1	20.0%	4	80.0%	
	Administration	32	71.1%	13	28.9%	
	Direct patient care	167	52.4%	152	47.6%	
	Indirect patient care (patient supportive office)	10	66.7%	5	33.3%	
How many hours per week do you work in your main job (on average)?	< 40	25	47.2%	28	52.8%	0.197
	40-50	145	57.8%	106	42.2%	
	51-60	37	48.7%	39	51.3%	
	> 60	12	42.9%	16	57.1%	
How many times do you wash your hands during a usual working day before the COVID 19 pandemic?	0-5 times per day	56	53.3%	49	46.7%	0.237
	6-10 times per day	67	47.5%	74	52.5%	
	11-20 times per day	52	58.4%	37	41.6%	
	more than 20 times per day	44	60.3%	29	39.7%	
How many times do you wash your hands during a usual working day during the COVID 19 pandemic?	0-5 times per day	14	56.0%	11	44.0%	0.738
	6-10 times per day	57	57.6%	42	42.4%	
	11-20 times per day	55	50.0%	55	50.0%	
	more than 20 times per day	93	53.4%	81	46.6%	
How much time do you spend on each hand washing before the COVID 19 pandemic?	Less than 40 seconds	87	48.3%	93	51.7%	0.110
	40-50 seconds	104	59.4%	71	40.6%	
	More than 50 seconds	28	52.8%	25	47.2%	
How much time do you spend on each hand washing during the COVID 19 pandemic?	Less than 40 seconds	35	56.5%	27	43.5%	0.790
	40-50 seconds	118	52.2%	108	47.8%	
	More than 50 seconds	66	55.0%	54	45.0%	
How many times do you use disinfecting alcohol for hands in a usual working day before the COVID 19 pandemic?	0-5 times per day	72	49.7%	73	50.3%	0.287
	6-10 times per day	60	50.8%	58	49.2%	
	11-20 times per day	53	58.9%	37	41.1%	
	more than 20 times per day	34	61.8%	21	38.2%	
How many times do you use disinfecting alcohol for hands in a usual working day during the COVID 19 pandemic?	0-5 times per day	16	64.0%	9	36.0%	0.655
	6-10 times per day	58	54.2%	49	45.8%	
	11-20 times per day	61	50.4%	60	49.6%	
	more than 20 times per day	84	54.2%	71	45.8%	

Risk factors for contact dermatitis among healthcare workers

Univariate logistic regression showed that contact dermatitis was significantly associated with several demographic characteristics and work conditions (Table 3). Female sex was associated with a higher prevalence (p=0.003). Participant age was also an important factor (p=0.019). Among the diverse occupational roles, medical interns were the most frequently affected group by contact dermatitis (p=0.038). A multivariate logistic regression analysis (Table 3), showed that female healthcare workers had 2.36 times the odds of reporting contact dermatitis than their male counterparts (95% CI: 1.23-1.23; p=0.008). Participants in the age group of 20-29 years with a mean of 26.47 years were more likely to develop contact dermatitis, compared to the other age groups (95% CI: 1.19-7.06; p=0.067). Pharmacists and interns had 3.69 and 4.90 times higher odds to have contact dermatitis than other occupations (95% CI: 0.95-7.33; 95% CI: 22.1; p=0.027, p=0.038, respectively). Those respondents who were involved in patient education and research activity at work were 6.48 (p=0.017) and 20.51 (p=0.024) times likely to develop contact dermatitis, compared to respondents with other roles (95% CI: 1.38-30.31; 95% CI: 1.49-282.15, respectively).

**Table 3:** Logistic regression models for occupational contact dermatitis * Not the reference category a: unadjusted (crude) OR b: complete model: multinomial multivariate logistic regression

Variables (n=408)		Model 1^a^ OR (95% CI)	p-value	Model 2^b^ OR (95% CI)	p-value
Gender	Female	1.88 (1.23 - 1.23)	0.003*	2.36 (1.25 - 4.45)	0.008*
	Male	Ref	Ref	Ref	Ref
Age	20 to 29	2.90 (1.19 -7.06)	0.019*	2.49 (0.93 - 6.63)	0.067
	30 to 39	1.96 (0.80 - 4.77)	0.137	2.14 (0.81 - 5.63)	0.120
	40 to 49	0.83 (0.30 - 2.26)	0.718	1.00 (0.34 - 2.92)	0.988
	> 49	Ref	Ref	Ref	Ref
Occupation	Doctors	1.36 (0.55 - 3.33)	0.496	1.93 (0.67 - 5.56)	0.219
	Nurses	1.41 (0.64 - 3.13)	0.386	1.20 (0.45 - 3.22	0.704
	Physiotherapists	0.54 (0.09 - 3.19)	0.501	0.71 (0.10 - 4.81)	0.732
	Pharmacists	2.64 (0.95 - 7.33)	0.062	3.69 (1.15 - 11.81)	0.027*
	Technicians	0.54 (0.15 - 1.92)	0.346	0.57 (0.13 - 2.34)	0.436
	Interns	4.90 (1.08 - 22.1)	0.038*	4.32 (0.79 - 23.46)	0.089
	Other	Ref	Ref	Ref	Ref
What is your major activity at work?	Patient education	3.33 (0.86 - 12.9)	0.082	6.48 (1.38 - 30.31)	0.017*
	Research	8.00 (0.69 - 91.7)	0.095	20.51 (1.49 - 282.15)	0.024*
	Administration	0.81 (0.23 - 2.84)	0.745	2.29 (0.54 - 9.75)	0.260
	Direct patient care	1.82 (0.60 - 5.44)	0.28	3.04 (0.84 - 10.97)	0.089
	Indirect patient care (patient supportive office)	Ref	Ref	Ref	Ref
Have your eyes ever shown allergic symptoms Itching, Watery, red or swollen eyes, e.g., from pollens or animals?	No	0.82 (0.29 - 2.33)	0.719	0.93 (0.31 - 2.84)	0.911
	Yes	2.28 (0.73 - 7.08)	0.152	3.03 (0.89 - 10.27)	0.074
	Don't know	Ref	Ref	Ref	Ref

## Discussion

Our primary goal of this study was to estimate the prevalence and investigate the risk factors for occupational-related contact dermatitis among healthcare workers in Al'Qassim Region, Saudi Arabia, during the Covid-19 pandemic.

Our study shows that the commonly reported symptom is skin dryness (92.9%), whereas an Ethiopian study was done in 2018 showed the majority of the participants reported skin redness (28.6%) [5]. However, the hands are the most affected area in both studies. Moreover, both studies show that nurses are more likely to suffer from skin changes than any other healthcare provider. It can be due to the fact that the majority of the participants were nurses, and nurses have more direct contact with patients. A study published in 2020 stated that an increase in hand hygiene could lead to skin changes involving skin dryness [3]. The pandemic undoubtedly has increased the necessity of frequent hand hygiene, thus increasing the unwanted skin changes.

Our study shows that the majority (76%) of healthcare workers wear latex gloves. Surprisingly, another study done during the pandemic in Hubei Province’s hospitals found the adverse effects of latex gloves on the skin [8]. Our results show that the most commonly reported symptom is skin dryness (92.9%). Similarly, the Hubei study results reported the most common symptom is skin dryness (55.8%). Moreover, both of these studies show the second most commonly reported symptom is skin itchiness - 50% and 31.2%, respectively. Skin itchiness could be an undiagnosed latex allergy since it is one of the symptoms of the disease. A study done in 2014 claims that healthcare providers are at high risk of developing latex allergy due to the increase in using latex gloves, the duration of wearing latex gloves, and exposure to the high concentration of latex antigens [9]. Our study shows that plastic gloves can cause less skin irritation than any other type of gloves (12%), whereas the Hubei study shows that none had reported any skin irritation when wearing plastic gloves [8].

Interestingly, our results show that females are more prone to develop contact dermatitis. Nevertheless, a study done in Singapore during severe acute respiratory syndrome (SARS) showed no gender predisposition, but another study published in 2016 claims that females are at a higher risk of developing facial and non-facial contact dermatitis [10, 11]. Moreover, our study shows that pharmacists and medical interns have a higher chance of developing contact dermatitis. On the other hand, the Singaporean study shows no occupation would be at a higher risk. We included in our study 58.6% of nurses, 16.2% of doctors, 8.3% of pharmacists, 4.9% of technicians, 2.9% of medical interns, 5.9% of patient education employees, and 1.2% of researchers, but they only included 14.3% of doctors, 73% of nurses, and 12.7% of clinic assistants and counter clerks. It is hard to conclude which occupation is at higher risk of developing skin changes during the pandemic due to the limitation of having equal representative numbers from each occupation. Our results and their results show that the young age group is more susceptible to developing contact dermatitis with a mean age of 26.4 years and 28.7 years, respectively. A study done in Riyadh states that elderly workers are less likely to have allergic contact dermatitis due to the age-related decrease in their inflammatory response [12]. 

Our study has some limitations. It does not cover all the hospitals, including private hospitals, in the Al'Qassim region. Also, not all the participants completely filled the questionnaire.

## Conclusions

Personal protective equipment has been markedly used during the COVID-19 pandemic, which led to skin manifestations among healthcare workers. In this study, we explored the prevalence and risk factors for occupational contact dermatitis among healthcare workers in Saudi Arabia. Our study showed risk factors for developing contact dermatitis among health care workers, which include a personal history of eye allergies, female gender, and young age. Also, skin symptoms have increased during the COVID-19 pandemic. The most commonly reported skin irritant was hand cleanser, followed by disinfectants and protective gloves. Among the healthcare workers, skin dryness was the highest prevalence, with 92.9%.
